# Supplementary material for: Dynamic morphological transformations in soft architected materials via buckling instability encoded heterogeneous magnetization
Source: Nat Commun. 2022 Dec 6;13:7514. doi: 10.1038/s41467-022-35212-6 (PMC9727123; doi:10.1038/s41467-022-35212-6)
Supplement: Supplementary file 1 — Supplementary Information [file 41467_2022_35212_MOESM1_ESM.pdf]

# Dynamic morphological transformations in soft architected materials via buckling instability encoded heterogeneous magnetization

Neng Xia[1], Dongdong Jin[1*ǂ], Chengfeng Pan[1], Jiachen Zhang[2], Zhengxin Yang[1], Lin Su[1], Jinsheng Zhao[1], Liu Wang[3], and Li Zhang[1,4,5,6*]

[*] Corresponding authors

[ǂ]Present address: School of Materials Science and Engineering, Harbin Institute of Technology (Shenzhen), Guangdong, China.

E-mail: jindongdong@link.cuhk.edu.hk lizhang@mae.cuhk.edu.hk (L. Zhang)

**The PDF file includes:**

Supplementary Note 1 Analytical model
Supplementary Note 2 Pumping efficiency
Supplementary Note 3 Magnetic field generation for the actuation of the untethered swimming robot
Supplementary Fig. 1. Swelling and deswelling processes of magneto-elastomer in toluene and ethanol
Supplementary Fig. 2. Swelling ratios of magneto-elastomers in different solvents
Supplementary Fig. 3. Magnetic hysteresis of magneto elastomers
Supplementary Fig. 4. Variation of deformation amplitude of the magneto-elastomer with swelling time in toluene
Supplementary Fig. 5. The quantitative relationship between wavelength, amplitude of the buckling configuration in toluene and the geometric parameters of the structure
Supplementary Fig. 6. Impact of magnetic field strength and direction angle on the wavelength
Supplementary Fig. 7. Magnetic responsive behaviors of magneto-elastomers with different connectivity types (distance: 8 mm)
Supplementary Fig. 8. Impact of four different connectivity types on the buckled configuration in the absorption of solvent (distance: 4mm)
Supplementary Fig. 9. Multiple transformation results of strips with different connectivity types and lattice structures
Supplementary Fig. 10. Impact of different connectivity types on the geometric transformation
Supplementary Fig. 11. Mixed deformation mode for $L/H$ = 7.41 ($L$ = 20 mm, $H$ = 2.7 mm).
Supplementary Fig. 12. Magnetic field simulation for the cuboid permanent magnet (size: 45×45×20 mm)
Supplementary Fig. 13. Deformation of lattice structures under different stimulation sequences
Supplementary Fig. 14. Fabricated hexagonal lattice, staggered square lattice, square and triangular lattices with pre-designing artificial defects and their morphological transformations by the stimuli from solvent and magnetic field
Supplementary Fig. 15. Geometric transformation of strip structures with cut patterns under solvent stimulation
Supplementary Fig. 16. Magnetic field simulation for the sphere permanent magnet (diameter: 35 mm) used for actuation
Supplementary Fig. 17. The phase diagram shows the fluid flow pattern induced by different magnetic field strengths and rotating frequency of the applied magnetic field
Supplementary Fig. 18. The impacts of magnetic field strength and rotating frequency on the measured maximum fluid velocity
Supplementary Fig. 19. Diverse dynamic flow field generated by the transformation of strip structures

Supplementary Fig. 20. Dynamic flow field generated by the transformation of two kinds of lattice structures
Supplementary Fig. 21. Demonstration of in-situ particle manipulation with the morphological transformation of the micro cellular structure.
Supplementary Fig. 22. Fluid mixing at low Re using the dynamic transformation of the magneto-elastomers
Supplementary Fig. 23. Side view of the deformation of the robotic structure during the actuation
Supplementary Fig. 24. Theoretical results and fitting for the relationship between $W_{\mathrm{nc}}$ and the aspect ratio (*L/H*).
Supplementary Table 1. Comparison of the pumping efficiency with literature
Supplementary References

**Other Supplementary Materials for this manuscript include the following:**

Supplementary Movie 1. Dynamic transformation of strip structure
Supplementary Movie 2. Coupling stimulation on the strip structure
Supplementary Movie 3. Morphological transformation of square lattice
Supplementary Movie 4. Fluidic behaviors induced by strip structures
Supplementary Movie 5. Reversible fluidic manipulation
Supplementary Movie 6. Merging and transporting of droplet
Supplementary Movie 7. Selective particle trap and release
Supplementary Movie 8. Sensitivity-enhanced biomedical analysis
Supplementary Movie 9. Efficient mixing of viscous fluid
Supplementary Movie 10. Untethered swimming robot

**Supplementary Note 1 Analytical model**

The analytical modeling of this work including (1) swelling-induced buckling behaviors, (2) buckling encoded 3D heterogeneous magnetization profile, and (3) magnetic response behavior.

(1) Swelling-induced buckling behaviors:

A thin elastomer strip has length *L*, height *H*, and thickness *T* as shown in Fig. 3. The bottom (*z* = 0) and top (*z* = *H*) edge of the elastomer strip are fixed and free, respectively. Foppl-von Karman equations were used to describe the deformation of thin flat plates [1,2], as shown in Eqs. (1) and (2).

$$\delta(y,z) = \frac{A}{2} \times f(z) \times sin\left(\frac{2\pi}{\lambda}y\right) \tag{1}$$

$$\frac{\partial^4\delta}{\partial y^4} + 2\frac{\partial^4\delta}{\partial y^2\partial z^2} + \frac{\partial^4\delta}{\partial z^4} + \frac{t_{11}}{D}\frac{\partial^2\delta}{\partial y^2} = 0 \tag{2}$$

$\delta(y,z)$ represents the out-of-plane displacement of the strip. $u_y(y,z)$ and $u_z(y,z)$ are the in-plane displacement along the *y* and *z* direction, respectively. $\lambda$ represents the wavelength. $t_{11}$ and *D* are the membrane force along the y direction and bending stiffness, respectively. *A* is the peak-to-peak value. $f(z)$ represents a normalized function, where $f(H) = 1$. By solving the ordinary differential equation for *f*(*z*), a general solution can be obtained as Eq. (3).

$$f(z) = C_1e^{-\alpha z} + C_2e^{\alpha z} + C_3cos(\beta z) + C_4sin(\beta z) \tag{3}$$

$$\alpha = \sqrt{\sqrt{\frac{t_{11}}{D}\left(\frac{2\pi}{\lambda}\right)^2} + \left(\frac{2\pi}{\lambda}\right)^2} \tag{4}$$

$$\beta = \sqrt{\sqrt{\frac{t_{11}}{D}\left(\frac{2\pi}{\lambda}\right)^2} - \left(\frac{2\pi}{\lambda}\right)^2} \tag{5}$$

The values of $C_1$, $C_2$, $C_3$, $C_4$ can be determined by the boundary conditions shown in Fig. 3. For the strip structure in Fig. 3a, the clamped edge and the free edge of the strip determine the equations:

$$\begin{cases} f(0) = 0 \\ \left.\frac{\partial f}{\partial z}\right|_{(z=0)} = 0 \\ \left.\left(\frac{\partial^2\delta}{\partial z^2} + \frac{v\partial^2\delta}{\partial y^2}\right)\right|_{(z=H)} = 0 \\ \left.\left[\frac{\partial^3\delta}{\partial z^3} + (2-v)\frac{\partial^3\delta}{\partial y^2\partial z}\right]\right|_{(z=H)} = 0 \end{cases} \tag{6}$$

, where $v$ is the Poisson's ratio. For the strip structure in Fig. 3b, the free-to-rotate junctions determine the equations.

$$\begin{cases} \delta(0,z) = \delta(L,z) = 0 \\ \left.\left(\frac{\partial^2\delta}{\partial y^2} + \upsilon\frac{\partial^2\delta}{\partial z^2}\right)\right|_{(y=0)} = \left.\left(\frac{\partial^2\delta}{\partial y^2} + \upsilon\frac{\partial^2\delta}{\partial z^2}\right)\right|_{(y=L)} = 0 \end{cases} \tag{7}$$

With the boundary conditions shown in Eq. (6), we can get the equations for $C_1$, $C_2$, $C_3$, $C_4$.

$$-\alpha C_1 + \alpha C_2 + \beta C_4 = 0 \tag{8}$$

$$C_1 + C_2 + C_3 = 0 \tag{9}$$

$$\mathbf{A}_{\text{co}} \begin{bmatrix} C_3 \\ C_4 \end{bmatrix} = \begin{bmatrix} A_{\text{co11}} & A_{\text{co12}} \\ A_{\text{co21}} & A_{\text{co22}} \end{bmatrix} \begin{bmatrix} C_3 \\ C_4 \end{bmatrix} = \begin{bmatrix} 0 \\ 0 \end{bmatrix} \tag{10}$$

$$Det\ (\mathbf{A}_{\text{co}}) = 0 \tag{11}$$

where,

$$A_{\text{co11}} = \left(\beta^2 + v\frac{4\pi^2}{\lambda^2}\right) cos(\beta H) + \left(\alpha^2 - v\frac{4\pi^2}{\lambda^2}\right) cosh(\alpha H)$$

$$A_{\text{co12}} = \left(\beta^2 + v\frac{4\pi^2}{\lambda^2}\right) sin(\beta H) + \beta\left(\alpha - v\frac{4\pi^2}{\alpha\lambda^2}\right) cosh(\alpha H)$$

$$A_{\text{co21}} = \left(-\beta^3 + (2 - v)\beta\frac{4\pi^2}{\lambda^2}\right) sin(\beta H) + \left(\alpha^3 - (2 - v)\alpha\frac{4\pi^2}{\lambda^2}\right) sinh(\alpha H)$$

$$A_{\text{co22}} = \left(\beta^3 + (2 - v)\beta\frac{4\pi^2}{\lambda^2}\right) cos(\beta H) + \left(\alpha^2\beta - (2 - v)\beta\frac{4\pi^2}{\lambda^2}\right) cosh(\alpha H)$$

As shown in Eq. (10), linear homogeneous equations determine the relationship between $C_3$ and $C_4$. To obtain nonzero solution for the equations, the determinant of the coefficient matrix is equal to zero, i.e., *Det (*$\mathbf{A}_{\text{co}}$*)*=0, which gives the relationship between $t_{11}$ and $\lambda$ and determines the critical swelling condition.

Firstly, we analyze the case with free-to-rotate junctions (shown in Fig. 3b). The relationship between $\lambda$ and $L$ can be determined by $W_{\text{n}} = L/\lambda_{\text{n}}$, where $2W_{\text{n}}$ is an integer and represents the deformation mode of strip with the junctions. By giving the value of $W_{\text{n}}$ and solving Eq (11), we can calculate the critical force $t_{11}^{\text{c}}$, and the relationship between the normalized critical force ($\frac{H^2 t_{11}^{\text{c}}}{ET^3}$) and geometry parameters, as shown in Fig. 3f. In the theoretical study, we fix the values of *H* and *T*, and study the impact of aspect ratio (*L/H*) on the deformation mode $W_{\text{n}}$. The theoretical and experimental results show that the relationship between *L/H* and $W_{\text{n}}$ is in a form of step function. When the *L/H* reaches a critical value, the mixed deformation modes would be exhibited as shown in Figs. 3f and 3g (*L/H* = 3.70, *L/H* = 7.41). Mixed deformation mode results for other critical conditions can be verified by extensive swelling tests.

Secondly, we analyze the case in Fig. 3a. By solving the Eq. (11) numerically under given $\lambda$, we can calculate the value of $t_{11}$ and gives the relationship between $\lambda$ and $t_{11}$. We still use the parameter $W_{\text{n}} = L/\lambda_{\text{n}}$to illustrate the deformation of the strip. While, in this case, $W_{\text{n}}$ can be any positive real number. As shown in Fig. 3f, for a given $W_{\text{n}}$, there is a nonlinear relationship between *L/H* and normalized critical force. The critical deformation mode $W_{\text{nc}} = L/\lambda_{\text{nc}}$ can be calculated according to the extreme point of the curve. Therefore, we could obtain the relationship between $W_{\text{nc}}$ and *L/H*, as shown in Supplementary Fig. 24, which shows a linear correlation between $W_{\text{nc}}$ and *L/H*. Through a linear fitting, $\lambda_{\text{nc}}$ can be obtained, as shown in Eq. (15), which demonstrates that the length of the strip has negligible effect on the wavelength of the buckled strip. With the value of $\lambda$ and $t_{11}$, the results of parameters including $C_1$, $C_2$, $C_3$, $C_4$, $\alpha$, and $\beta$ can be obtained.

$$\lambda_{\text{nc}} \approx 3.256H \tag{15}$$

The amplitude of the out-of-plane displacement ($\delta$) can be obtained via the minimization of the total energy [1], as shown in Eqs. (16) and (17).

$$U = \frac{0.05ET^3A^2}{H^2(1-v^2)} - \frac{0.0434ETA^2(\chi-1)}{\chi H(1-v^2)} + \frac{0.3745ETH(\chi-1)^2}{\chi^2(1-v^2)} + \frac{0.0029ETA^4}{H^3(1-v^2)} \tag{16}$$

$$\frac{\partial U}{\partial A} = 0 \tag{17}$$

$U$ represents the total energy of the strip and $\chi$ represents the swelling ratio. The values of the peak-to-peak amplitude ($A$) can be calculated.

$$A = 2.724H\sqrt{\frac{\chi-1}{\chi} - 1.155\frac{T^2}{H^2}} \tag{18}$$

Experimental results were employed to validate the theoretical model of the buckled strip, as shown in Fig. 3c and 3d. The agreement between the experimental and theoretical results validates the model which could quantitatively describes the relationship between the buckling results and the geometry parameters.

(2) Buckling encoded 3D heterogeneous magnetization profile:

A theoretical model was developed to describe the formation of 3D heterogeneous magnetization profile. As shown in Fig. 3e, $\mathbf{M}'(x+\delta, y+u_y, z+u_z)$ and $\mathbf{M}(x,y,z)$ represent magnetization vectors under deformation and after recovery, respectively. Considering the recovery process, we can derive the relationship between $\mathbf{M}'$ and $\mathbf{M}$, as shown in Eq. (19).

$$\mathbf{M}(x,y,z) = \frac{\mathbf{R}}{J}\mathbf{M}'(x+\delta, y+u_{\mathrm{y}}, z+u_{\mathrm{z}}) \tag{19}$$

$J = det(\mathbf{F})$, where **F** is the deformation gradient of an element of body shown in Fig. 3e. **R** is the rotational component of **F**. To estimate the magnetization vector after recovery, we focus on the rotation of the element along *z* axis and y axis with Eqs. (20)-(22).

$$\mathbf{R} = \begin{bmatrix} cos(\psi) & 0 & sin(\psi) \\ 0 & 1 & 0 \\ -sin(\psi) & 0 & cos(\psi) \end{bmatrix} \times \begin{bmatrix} cos(\theta) & -sin(\theta) & 0 \\ sin(\theta) & cos(\theta) & 0 \\ 0 & 0 & 1 \end{bmatrix} \tag{20}$$

$$\theta = arctan\left(\frac{\partial\delta}{\partial y}\right) = arctan\left\{\frac{\pi A}{\lambda}[C_1e^{-\alpha z} + C_2e^{\alpha z} + C_3cos(\beta z) + C_4sin(\beta z)]cos\left(\frac{2\pi}{\lambda}y\right)\right\}$$
(21)

$$\psi = arctan\left(\frac{\partial\delta}{\partial z}\right) = arctan\left\{\frac{A}{2}[-\alpha C_1e^{-\alpha z} + \alpha C_2e^{\alpha z} - \beta C_3sin(\beta z) + \beta C_4cos(\beta z)]sin\left(\frac{2\pi}{\lambda}y\right)\right\} \tag{22}$$

There is a uniform pulse magnetic field applied to the buckled strip. Thus, $\mathbf{M}' = M_0[1 \quad 0 \quad 0]^T$, where $M_0$ represents the magnitude of remanent magnetization. By combining Eqs. (19) and (20), we can obtain the expression of **M**, as shown in Eq. (23).

$$\mathbf{M} = \begin{bmatrix} M_{\mathrm{x}} \\ M_{\mathrm{y}} \\ M_{\mathrm{z}} \end{bmatrix} = M_0\begin{bmatrix} cos(\psi)cos(\theta) \\ sin(\theta) \\ -sin(\psi)cos(\theta) \end{bmatrix} \tag{23}$$

To validate the theoretical model, we employed a strip with $L$ = 28 mm, $H$ = 2.7 mm, $T$ = 0.5 mm. The variation of magnetization vector along the y direction at $z=0$ and $z=H$ is shown in Fig. 3h. The magneto-optical result in Fig. 3i demonstrates the distribution of the magnetic flux density which is consistent with the simulation result based on the calculated magnetization profile.

(3) Magnetic response behavior:

To elucidate the impact of external magnetic field on the deformation of the magnetic elastomer, a theoretical model to illustrate the relationship between magnetic torque and out-of-plane displacement ($\delta_{\mathrm{m}}$) was established. The out-of-plane displacement of the strip show in Fig. 3e can be calculated using plate theory for thin plate, as shown in Eqs. (24) and (25).

$$M_{\mathrm{z}} = D\left(\frac{\partial^2 \delta_{\mathrm{m}}}{\partial z^2} + \upsilon \frac{\partial^2 \delta_{\mathrm{m}}}{\partial y^2}\right) = \iiint_V (-\tau_{\mathrm{m}})\, dV \quad (24)$$

$$\tau_{\mathrm{m}} = [0 \quad 0 \quad 1]\mathbf{R}'\mathbf{M} \times \mathbf{B} \quad (25)$$

$\tau_{\mathrm{m}}$ is the magnetic torque and $M_{\mathrm{z}}$ represents the bending moment applied to the elastomer. $\mathbf{R}'$ is the rotational matrix for the change of magnetization direction during the deformation. $V$ represents the volume of the plate. To estimate the magnetic actuated deformation, we utilize small deflection assumption by approximating $\mathbf{R}'$ as an identity matrix, and assume that the effective body torque distribution along the elastomer strip is equivalent to that generated by external bending moment applied at the free end of the strip [3]. Furthermore, we express the out-of-plane displacement as $\delta_{\mathrm{m}}(y,z) = \Theta_{\mathrm{m}}(y) \times \Lambda_{\mathrm{m}}(z)$. With these simplifications, Eq. (26) can be obtained for the case that an external magnetic field is applied along the $x$ direction with magnitude of $B_0$.

$$D\left(\Theta_{\mathrm{m}} \frac{d^2 \Lambda_{\mathrm{m}}}{dz^2} + \upsilon \Lambda_{\mathrm{m}} \frac{d^2 \Theta_{\mathrm{m}}}{dy^2}\right) = \int_0^y \int_0^H \{M_0 B_0 T \times sin[\theta(y,z)]\}\, dydz \quad (26)$$

The function $sin[\theta(y,z)]$ can be fitted as: $sin[\theta(y,z)] \approx G(z) \times cos(ky + \emptyset)$. Based on the right side of Eq. (26), we can define $\Theta_{\mathrm{m}}$ as: $\Theta_{\mathrm{m}} = sin(ky + \emptyset)$. Thus, Eq. (26) can be written as:

$$D\left(\frac{d^2 \Lambda_{\mathrm{m}}}{dz^2} - \upsilon k^2 \Lambda_{\mathrm{m}}\right) sin(ky + \emptyset) = \int_0^H \left\{\frac{M_0 B_0 T}{k} \times G(z)\right\} dz \times sin(ky + \emptyset) \quad (27)$$

By solving Eq. (27) with the boundary condition ($\Lambda_{\mathrm{m}}(z=0) = 0$) numerically, we could obtain the result of $\delta_{\mathrm{m}} = \Lambda_{\mathrm{m}} \times sin(ky + \emptyset)$. We employed a strip with $L$ = 28 mm, $H$ = 4.3 mm, $T$ = 0.5 mm to validate the model, as shown in Fig. 3j. There is an agreement with the experimental result of the deformed strip.

In summary, we perform theoretical studies for the solvent and magnetic responsive behaviors of the magneto-elastomer. The theoretical models could give the quantitative relationship between the key parameters of buckling transformation and geometric parameters, the possible deformation modes of buckling structures, the quantitative description of 3D heterogeneous magnetization profiles, and the deformation under magnetic stimuli. The studies provide a theoretical guideline for the structural design and understanding of the underlying transformation mechanisms.

**Supplementary Note 2 Pumping efficiency**

To evaluate the pumping efficiency of fluid generation methods, a dimensionless parameter ($\varepsilon_{\mathrm{ef}}$) similar to references [4,5] is adopted as Eq. (28).

$$\varepsilon_{\mathrm{ef}} = \frac{\eta_{\mathrm{vis}} Q^2}{V_{\mathrm{vol}} P} \quad (28)$$

$\eta_{\mathrm{vis}}$ and $Q$ represent the viscosity of the pumping fluid and volume flow rate, respectively. $V_{\mathrm{vol}}$ is the volume of the magnetic elastomer used for pumping fluid and $P$ is the input power. The volume flow rate $Q$ = 25.6 μl/min of the proposed method is obtained by measuring the change

in mass of the fluid flowing from the outlet with the actuation frequency of 5 Hz [6]. Glycerol is adopted as the fluid with a viscosity $\eta_{\text{vis}}$ of 0.876 $N \cdot s/m^2$. The input power $P$ is determined by integrating the work performed by magnetic torque on the dynamic transformation of the magnetic elastomer [7], as shown in Eq. (29), where $\boldsymbol{\omega}(x, y, z, t)$ represent angular velocity and $T$ is the time for one actuation cycle.

$$P = \frac{1}{T}\int_0^T \int_{V_{vol}} |(\mathbf{R}'\mathbf{M} \times \mathbf{B}) \cdot \boldsymbol{\omega}|\, dVdt \tag{29}$$

Eq. (30) is used to calculate the kinematic property of the magnetic elastomer over one actuation cycle.

$$\delta_{\text{m}}(x, y, z, t) = \Lambda_{\text{m}}(z) \times sin\left(\frac{2\pi}{\lambda}y + \phi(t)\right) \tag{30}$$

$\Lambda_{\text{m}}$ is the magnitude of the out-of-plane displacement under magnetic actuation and $\phi(t)$ is the phase shift induced by the varying magnetic field direction and can be measured from experimental results. According to the analysis of magnetic response behavior, the rotational deflection and angular velocity can be obtained as Eqs. (31) and (32).

$$\alpha = arctan\left(\frac{\partial\delta_{\text{m}}}{\partial y}\right) = arctan\left\{\frac{2\pi\Lambda_{\text{m}}}{\lambda} cos\left[\frac{2\pi}{\lambda}y + \phi(t)\right]\right\} \tag{31}$$

$$\omega(x, y, z, t) = \frac{\partial\alpha}{\partial t} = \frac{-2\pi\cdot\Lambda_{\text{m}}}{\lambda\left[1+\left(\frac{\partial\delta_{\text{m}}}{\partial y}\right)^2\right]} \cdot sin\left(\frac{2\pi}{\lambda}y + \phi(t)\right) \cdot \frac{d\phi(t)}{dt} \tag{32}$$

By applying a rotating magnetic field $\mathbf{B} = B_0\left[cos\left(\frac{2\pi t}{T}\right) \quad sin\left(\frac{2\pi t}{T}\right) \quad 0\right]^T$, the input energy can be calculated as Eq. (33).

$$P = \frac{1}{T}\int_0^T \int_{V_{\text{vol}}} \left|M_0 B_0 \cdot sin\left(\frac{2\pi t}{T} - \alpha - \theta\right) \cdot \omega\right| dVdt \tag{33}$$

By solving Eq. (33) numerically, we could obtain the average input power as 1.4×10$^{-3}$ W. The power done by magnetic torque contributes to the storage of strain energy, the variation of kinetic energy of the magnetic elastomer and fluid.

The comparison of the pumping efficiency $\varepsilon_{\text{ef}}$ with other methods is shown in Table S1, which demonstrates that the developed flow generation method outperforms most reported soft pumping device in terms of pumping efficiency.

## Supplementary Note 3 Magnetic field generation for the actuation of the untethered swimming robot

3-axis Helmholtz electromagnetic coils are used to generate a programmable magnetic field, as shown in Eq. (34). $\mathbf{B} = [B_{\text{x}} \quad B_{\text{y}} \quad B_{\text{z}}]^T$ represents the magnetic field used for the actuation of the swimming robot. $B_{\text{a}}$ and $B_{\text{c}}$ are constant and represent the magnitude of the magnetic field along the $x$ and $y$ direction, respectively. $f$ is the frequency of the applied magnetic field and $t$ is time. A custom-programmed software using LabVIEW is adopted to control the proposed magnetic field. By tuning the direction angle of the magnetic field, the untethered robot could swim along a given trajectory.

$$\mathbf{B} = \begin{bmatrix} B_{\text{x}} \\ B_{\text{y}} \\ B_{\text{z}} \end{bmatrix} = \begin{bmatrix} B_{\text{a}} \times sin(2\pi f t) \\ B_{\text{c}} \\ 0 \end{bmatrix} \tag{34}$$

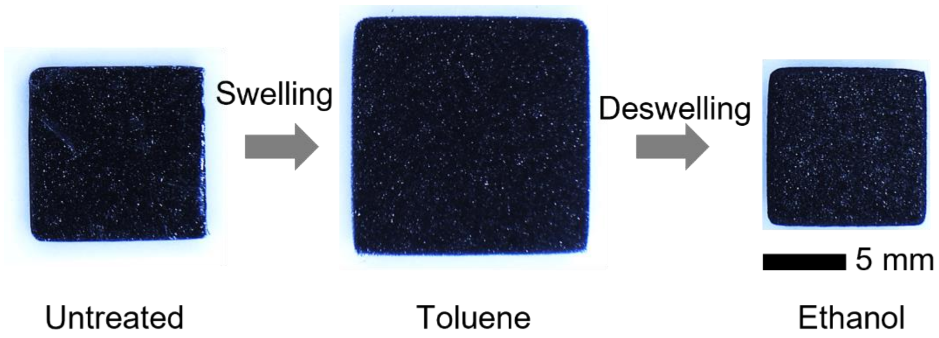


**Supplementary Fig. 1. Swelling and deswelling processes of magneto-elastomer in toluene and ethanol.** The scale bar is 5 mm.

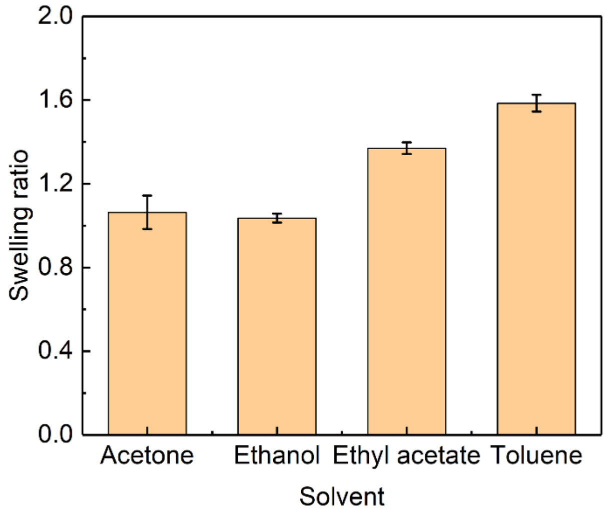


**Supplementary Fig. 2. Swelling ratios of magneto-elastomers in different solvents.** Error bars stand for the standard error of the mean with the number of trials $n$ = 3.

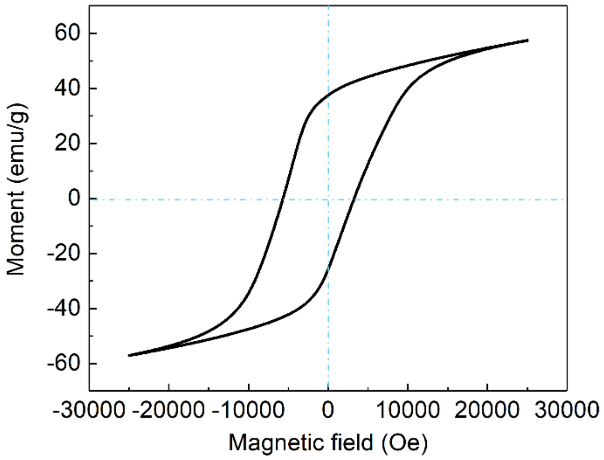


**Supplementary Fig. 3. Magnetic hysteresis of magneto elastomers.** Magnetic characterization result of the magneto-elastomer was tested by a PPMS model 6000 Quantum Design VSM with a maximum magnetic field strength of 25000 Oe.

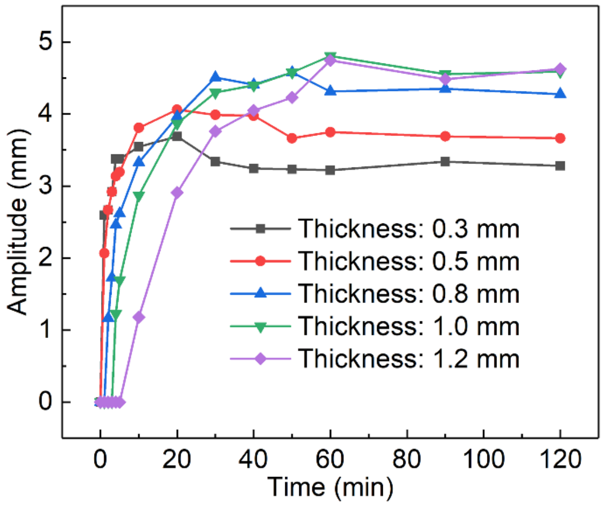


**Supplementary Fig. 4. Variation of deformation amplitude of the magneto-elastomer with swelling time in toluene.** The relationship between swelling time and deformation amplitude of magneto-elastomers with five different thicknesses.

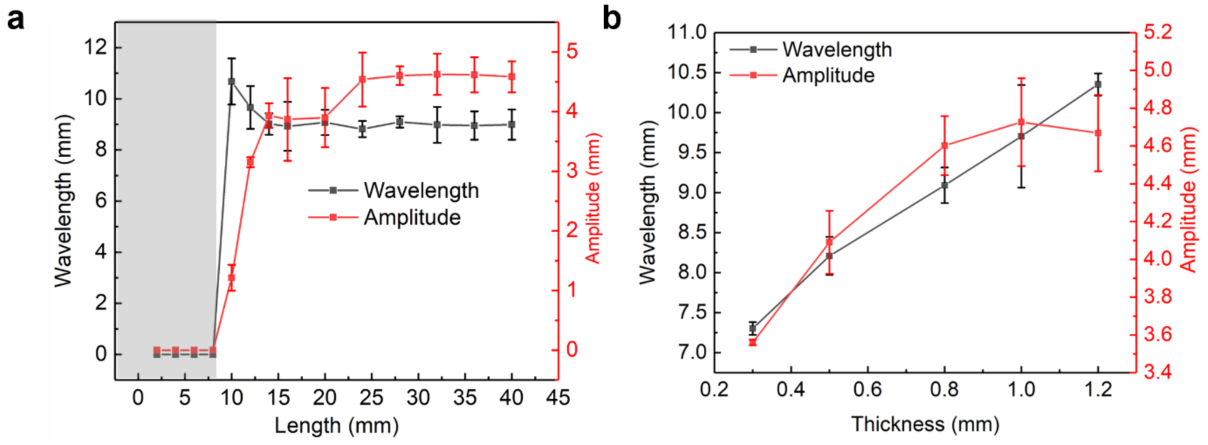


**Supplementary Fig. 5. The quantitative relationship between wavelength, amplitude of the buckling configuration in toluene and the geometric parameters of the structure including length (a) and thickness (b).** The gray area represents no wavy pattern generated in the elastomeric structure. Error bars stand for the standard error of the mean with the number of trials $n = 3$.

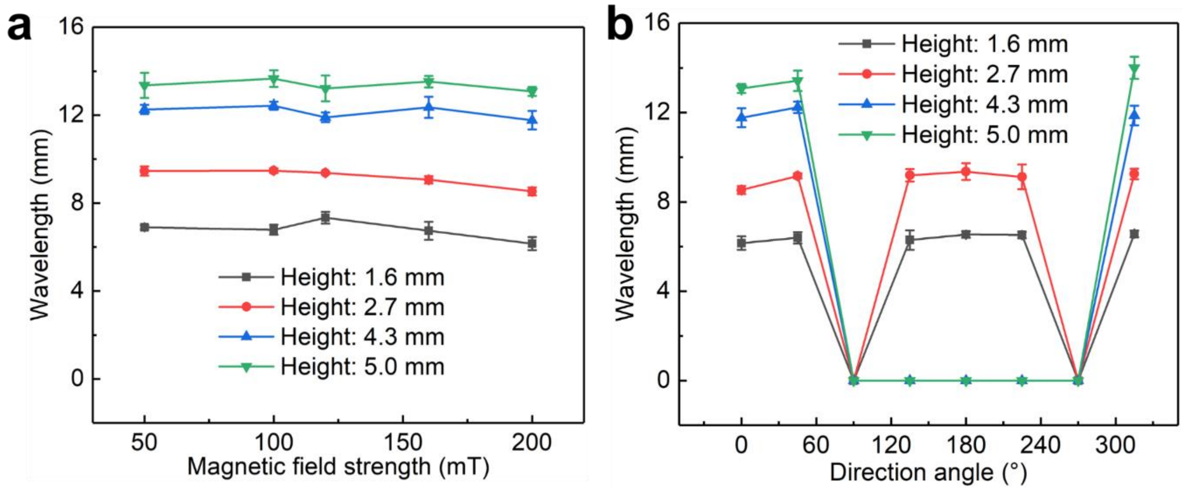


**Supplementary Fig. 6. Impact of magnetic field strength and direction angle on the wavelength. a** Effect of magnetic field strength on the wavelength. **b** Effect of direction angle on the wavelength. Buckling instability-encoded magnetized strip structures with different heights are fabricated for test. The wavelength of magneto-elastomer almost keeps constant under different magnetic field strength, indicating the magnitude of magnetic torque only affects the deformation amplitude rather than wavelength. On the other hand, in Fig. S6**b**, when the direction angle of magnetic field is between 90° and 270°, collapse or torsion of the magnetic strips may occur, especially for the strip structure with larger height, leading to the disappearance of wavy pattern. But once the wave forms in magneto-elastomers, it is found that the adjustment of magnetic field direction angle cannot induce any obvious change in the wavelength. Error bars stand for the standard error of the mean with the number of trials $n = 3$.

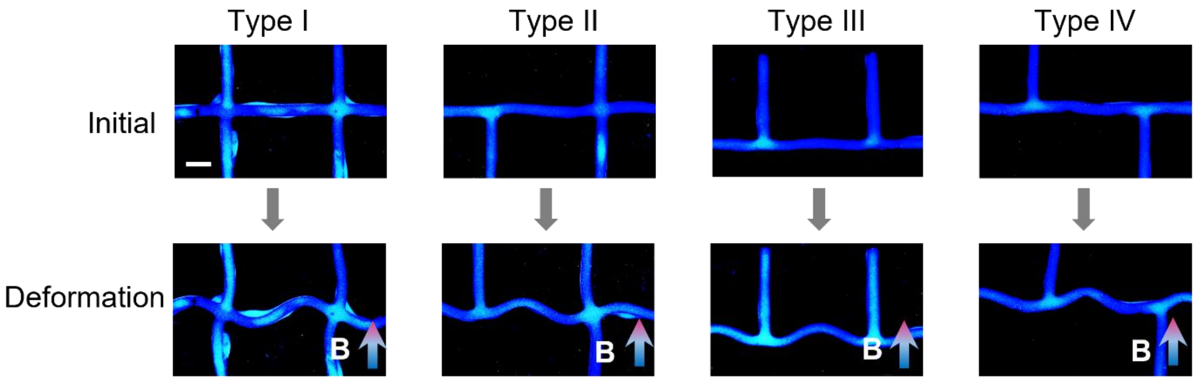


**Supplementary Fig. 7. Magnetic responsive behaviors of magneto-elastomers with different connectivity types (distance: 8 mm).** The scale bar is 2 mm.

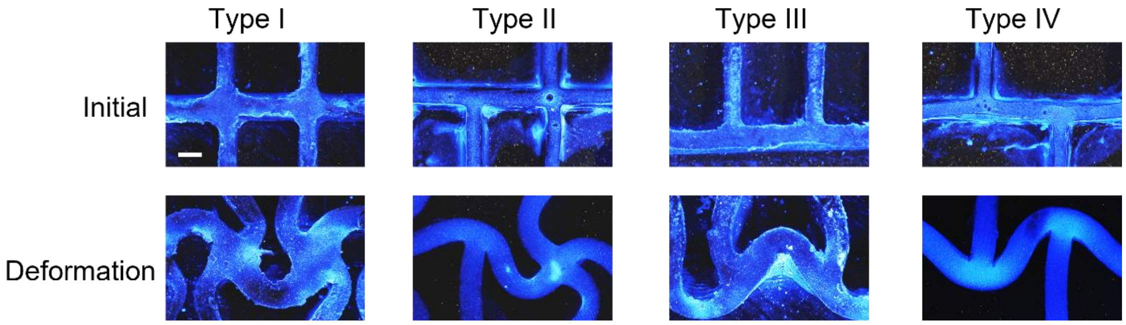


**Supplementary Fig. 8. Impact of four different connectivity types on the buckled configuration in the absorption of solvent (distance: 4mm).** The scale bar is 2 mm.

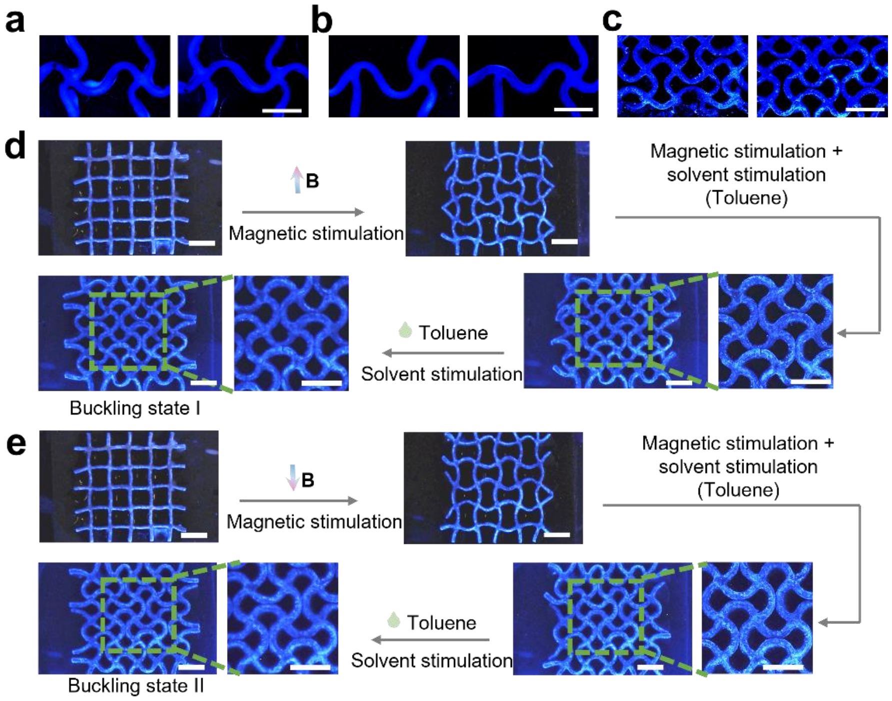


**Supplementary Fig. 9. Multiple transformation results of strips with different connectivity types and lattice structures. a-c** The dual transformation results of the elastomer structures; **d-e** Tuning the buckled state of square lattice via magnetic and solvent stimulation. By applying magnetic stimulation first followed by the solvent stimulation, the structure can form different buckling states with the change of the direction of the external magnetic field. The scale bars are 5 mm.

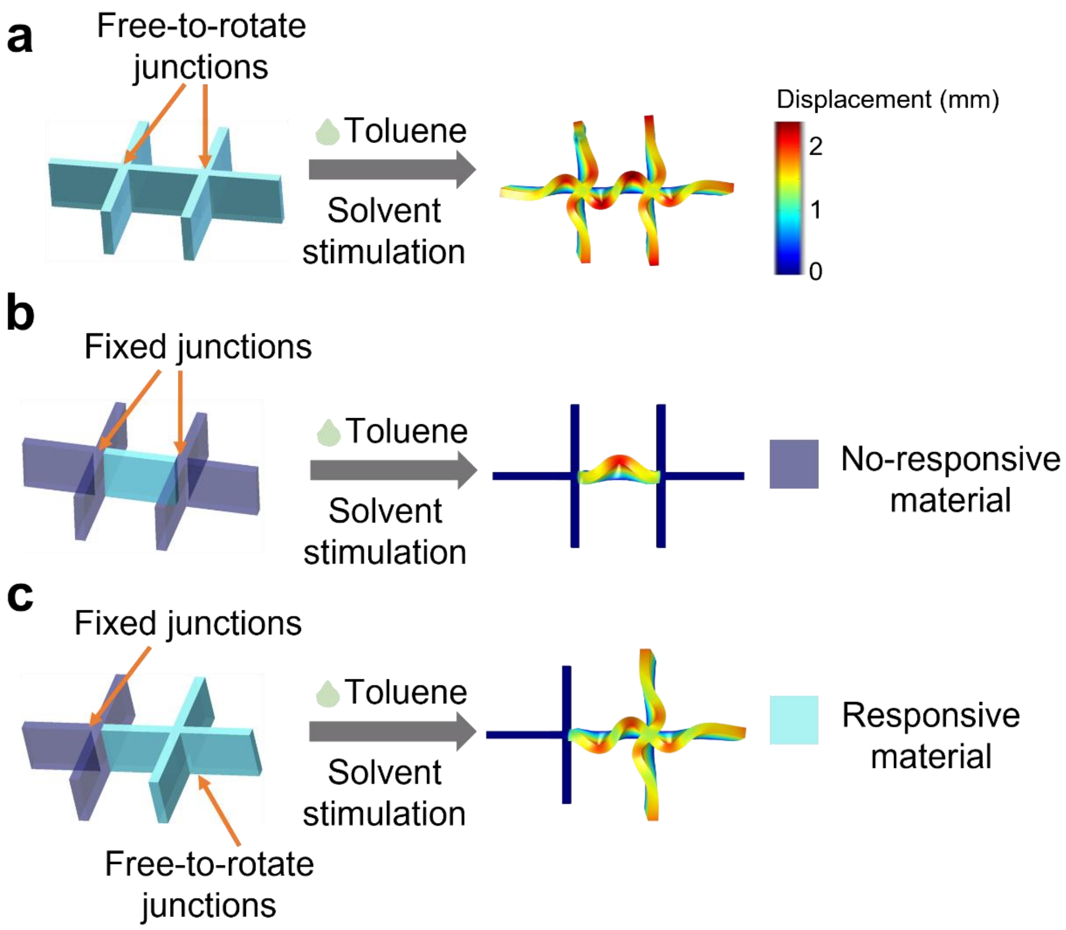


**Supplementary Fig. 10. Impact of different connectivity types on the geometric transformation. a** Strip structure with free-to-rotate junctions. **b** Strip structure with fixed junctions. **c** Strip structure with both fixed and free-to-rotate junctions. As shown in Fig. S18**b**, the two ends of the plate structure are distributed with non-responsive material and fixed constraints, which restricts the rotation at the ends. With such restrictions, the structure shows a bell-shaped deformation under solvent stimulation which is different from that with free-to-rotate junctions. Asymmetric deformation would be formed by adopting mix connectivity types.

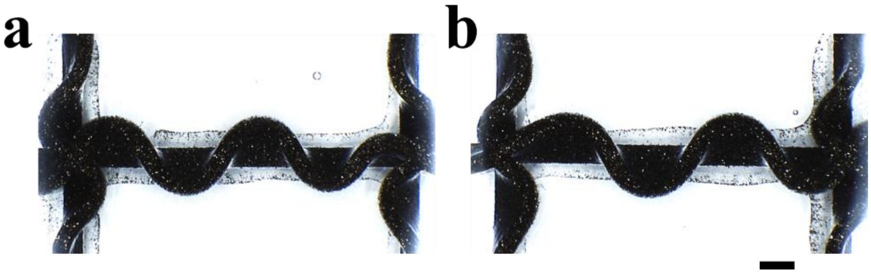


**Supplementary Fig. 11. Mixed deformation mode for *L/H* = 7.41 (*L*= 20 mm, *H*=2.7 mm).**
**a** $W_n$ = 2.5. **b** $W_n$ = 2.0. The scale bar is 2 mm.

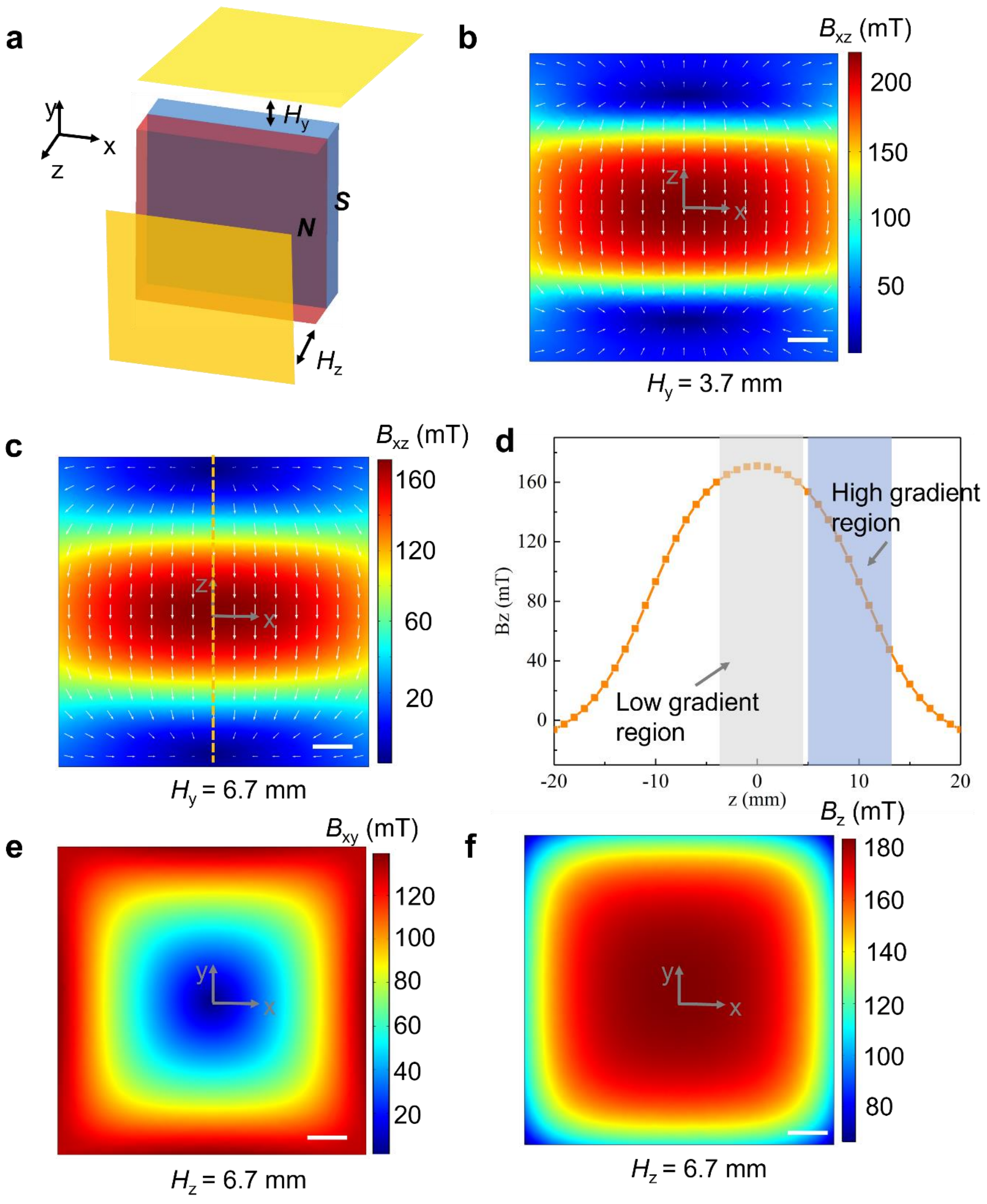


**Supplementary Fig. 12. Magnetic field simulation for the cuboid permanent magnet (size: 45×45×20 mm). a** Schematic of the cuboid permanent magnet. **b-c** Magnetic field distribution of the cuboid permanent magnet in x-z plane with $H_y$ = 3.7 and 6.7 mm. A uniform unidirectional magnetic field is generated in the central region of the x-z plane. The magnetic field distribution along the centerline in Fig. S12**c** is shown in Fig. S12**d**, which shows that large magnetic field gradient is formed in the edge regions of the permanent magnet. **e-f** Magnetic field distribution of the cuboid permanent magnet in x-y plane with $H_z$ = 6.7 mm. The scale bar is 5 mm.

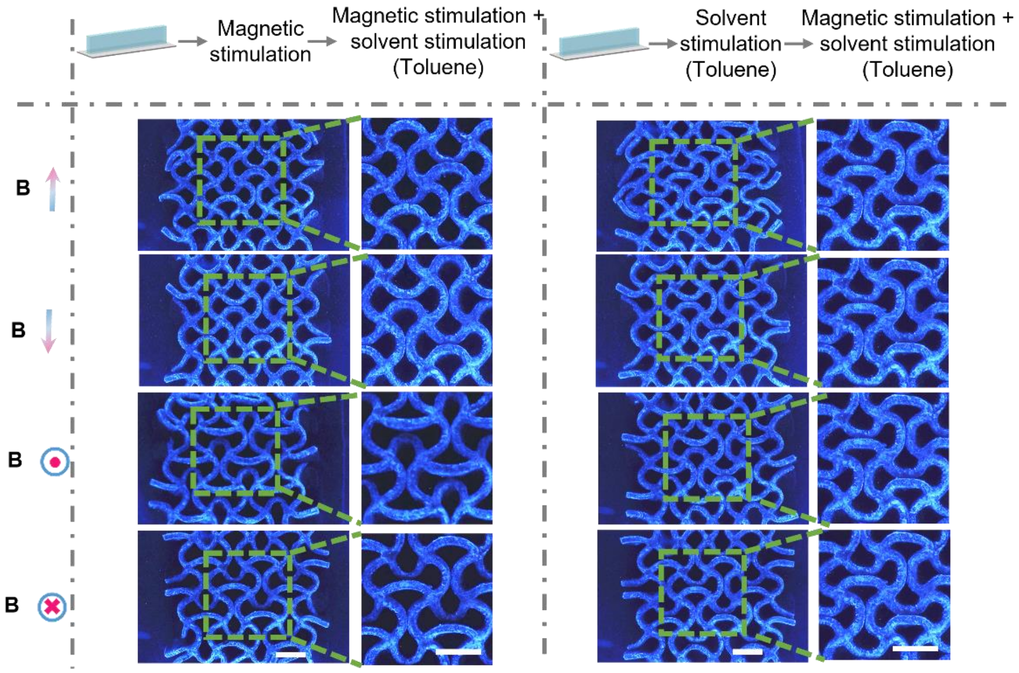


**Supplementary Fig. 13. Deformation of lattice structures under different stimulation sequences.** By applying magnetic stimulation first followed by the solvent stimulation, the structure can form different buckling states with the change of the direction of the external magnetic field. While for the case of applying solvent stimulation first, the external magnetic stimulation cannot provide sufficient energy to change its morphology. The scale bars are 5 mm.

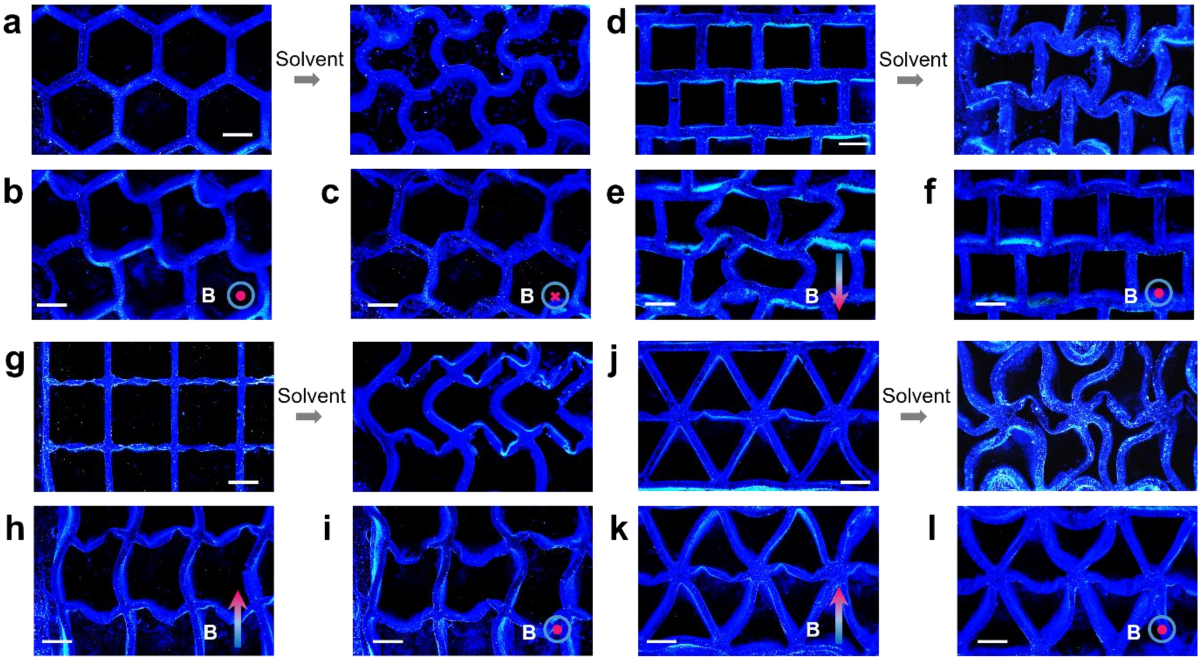


**Supplementary Fig. 14. Fabricated lattice structures and their morphological transformations by the stimuli from solvent and magnetic field. a-c** Hexagonal lattice. **d-f** **S**taggered square lattice. **g-i** Square lattices with pre-designing artificial defects. **j-l** Triangular lattices with pre-designing artificial defects. The scale bar is 2 mm.

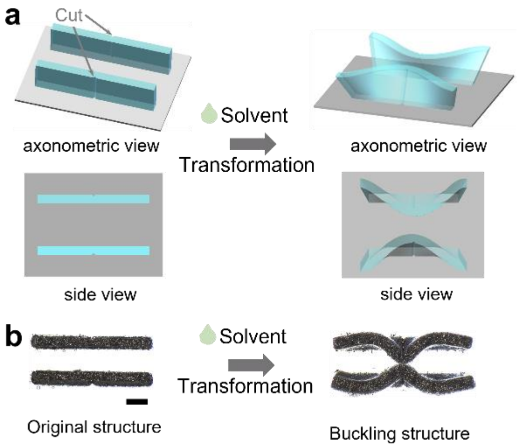


**Supplementary Fig. 15. Geometric transformation of strip structures with cut patterns under solvent stimulation. a** Schematic diagram of the cut patterns and transformation process. **b** Experimental results of the transformation of strip structure with cut patterns. The scale bar is 2 mm.

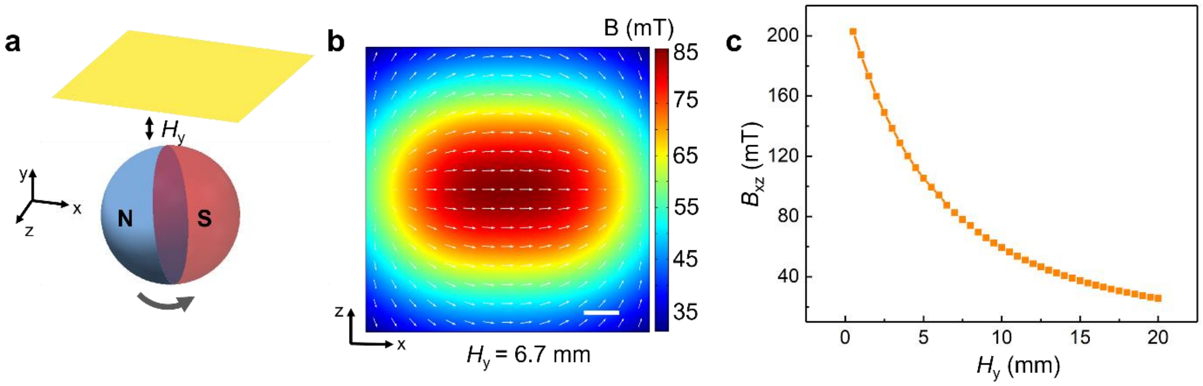


**Supplementary Fig. 16. Magnetic field simulation for the sphere permanent magnet (diameter: 35 mm) used for actuation. a** Schematic of the sphere permanent magnet. **b** Magnetic field distribution of the sphere permanent magnet in x-z plane with $H_y$ = 6.7 mm. **c** The change of magnetic field strength $B_{xz}$ with the distance $H_y$.

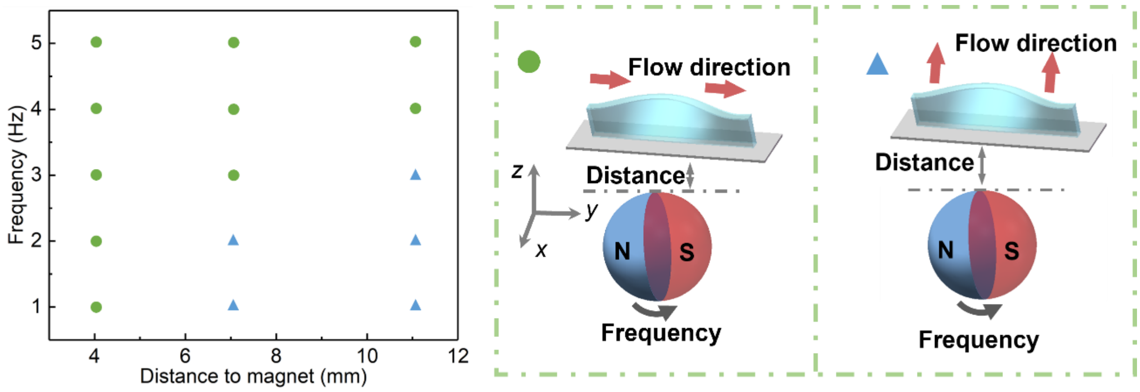


**Supplementary Fig. 17. The phase diagram shows the fluid flow pattern induced by different magnetic field strengths and rotating frequency of the applied magnetic field.** The magnetic field strength is tuned by the distance between the substrate and the sphere permanent magnet. Circular and triangular symbols represent different fluidic behaviors induced by the dynamic transformations.

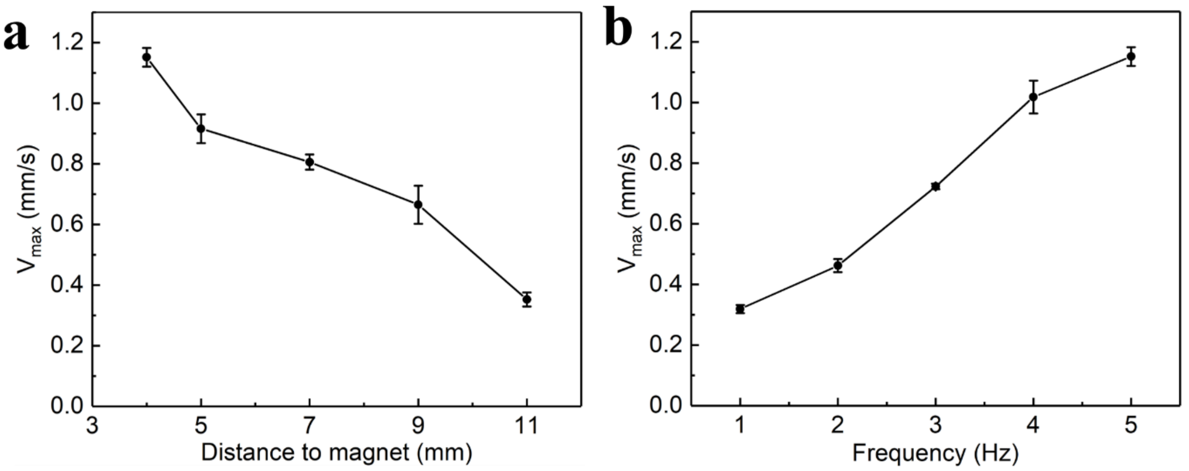


**Supplementary Fig. 18. The impacts of magnetic field strength (a) and rotating frequency (b) on the measured maximum fluid velocity.** Error bars stand for the standard error of the mean with the number of trials n = 3.

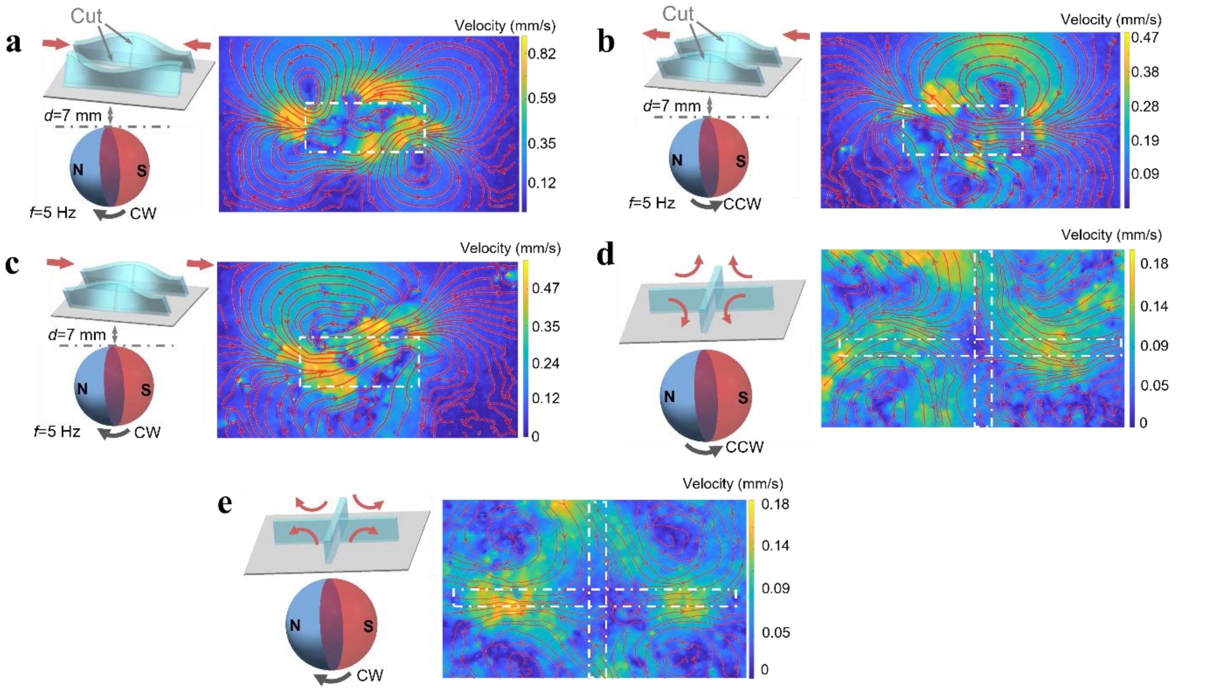


**Supplementary Fig. 19. Diverse dynamic flow field generated by the transformation of strip structures. a** Two strip structures with symmetric shape-morphing results induced by symmetric cuts arranged in strip structures. **b-c** Two strip structures with same shape-morphing results. **d-e** Elastomeric structure with criss-cross design.

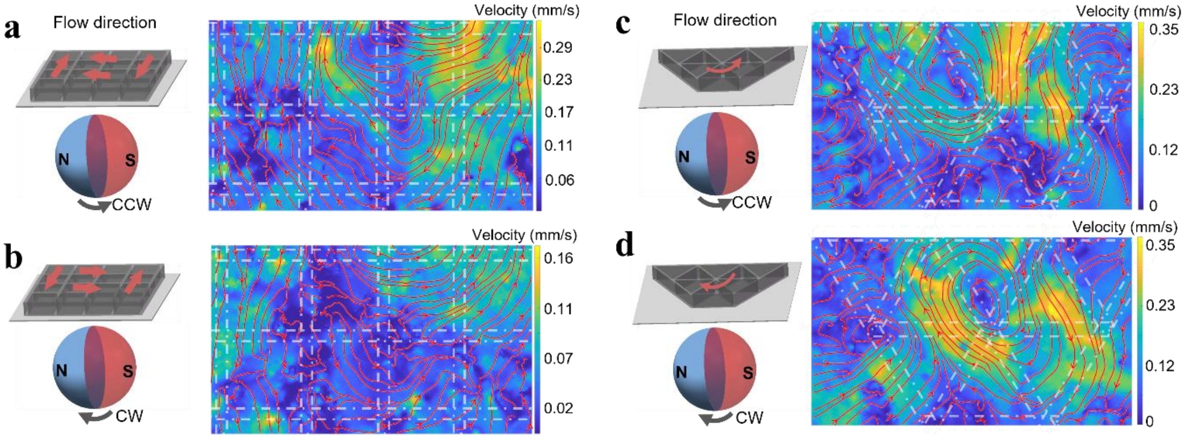


**Supplementary Fig. 20. Dynamic flow field generated by the transformation of two kinds of lattice structures. a-b** The dynamic flow field induced by square lattice. **c-d** The dynamic flow field induced by triangular lattice.

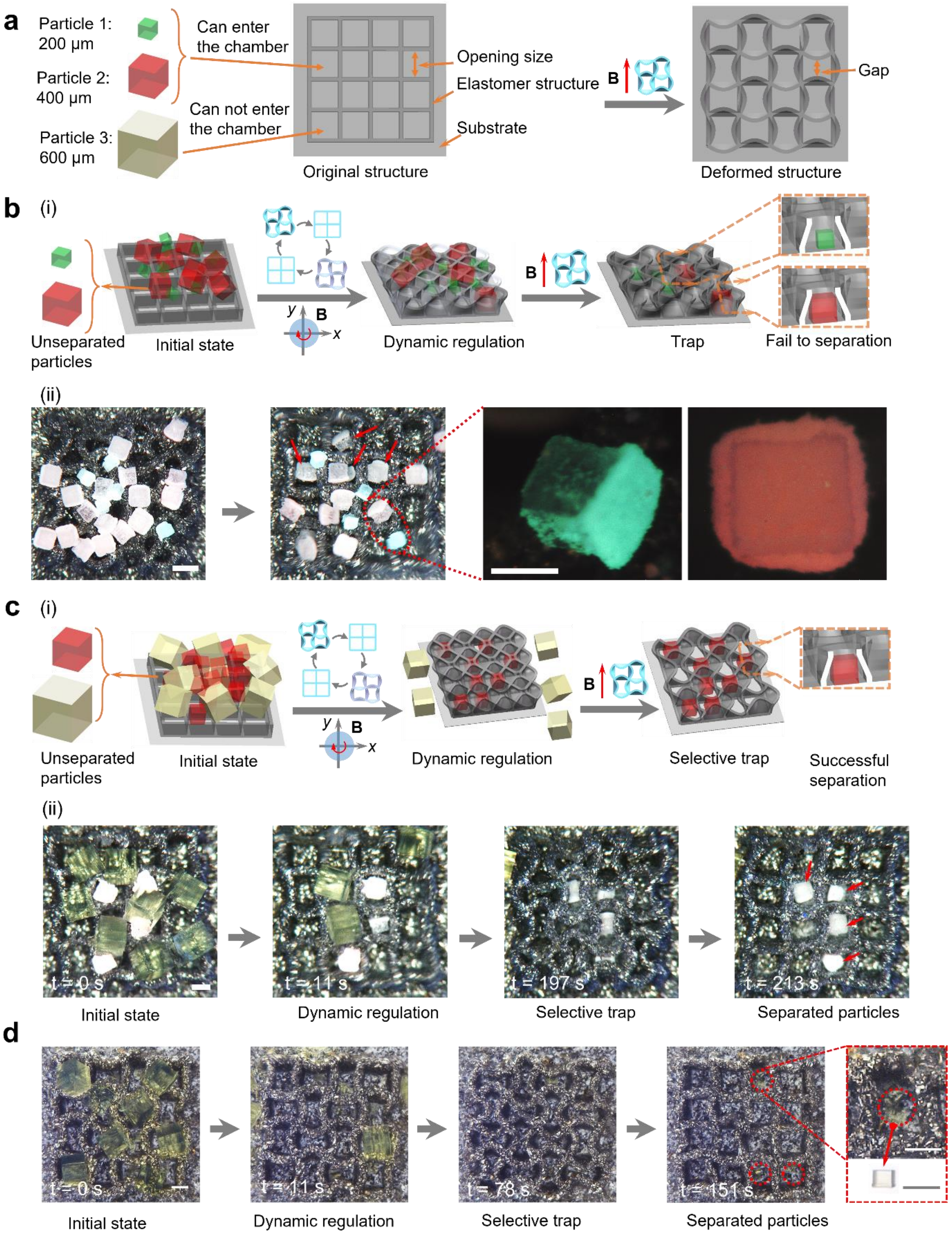


**Supplementary Fig. 21. Demonstration of in-situ particle manipulation with the morphological transformation of the micro cellular structure. a** Schematic illustration of initial and deformed states of the micro cellular structure and the unseparated microparticles. The micro cellular structure has an opening size of 500 µm, which allows the reservation of 200 µm (green) and 400 µm (red) particles in the chamber and the extrusion of 600 µm (yellow) particles. By applying magnetic stimulus, the deformed cellular structure with a gap would be formed whose size can be regulated by the magnetic field strength. **b** All unseparated particles

are smaller than the opening size (e.g., 200 μm particles and 400 μm particles), effective separation cannot be achieved through the dynamic structural transformation. **c** Selective particle trapping through the dynamic structural transformation. Particles of 400 (red) and 600 μm (yellow) in size are adopted. **d** Selective particle trapping through the dynamic structural transformation. Particles of 200 and 600 μm in size are adopted. The scale bars in **b-c** are 200 μm. The scale bars in **d** are 400 μm.

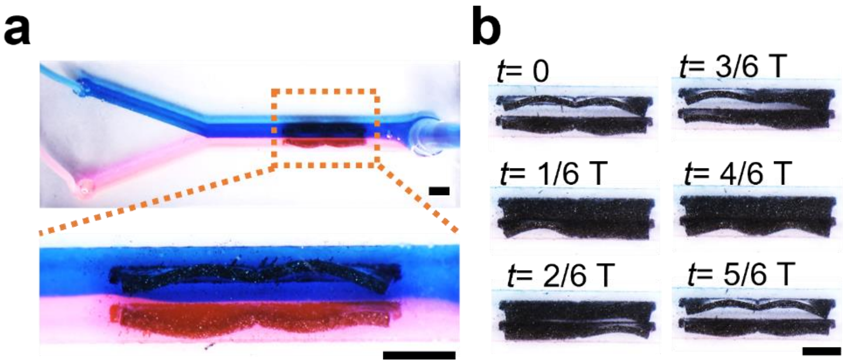


**Supplementary Fig. 22. Fluid mixing at low Re using the dynamic transformation of the magneto-elastomers. a** Images of the fluid mixing device including glycerol layers with two colors and magneto-elastomers. **b** Dynamic transformation of the magneto-elastomers in the fluidic channel under rotating magnetic field. The scale bars are 3 mm.

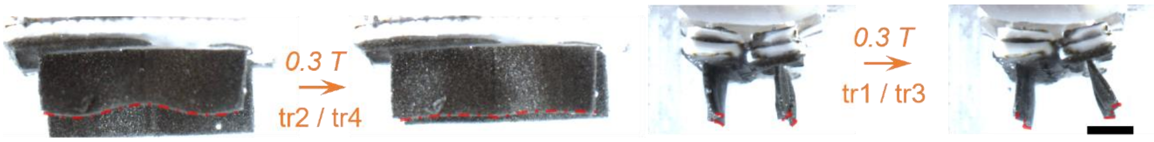


**Supplementary Fig. 23. Side view of the deformation of the robotic structure during the actuation.** The scale bars are 5 mm.

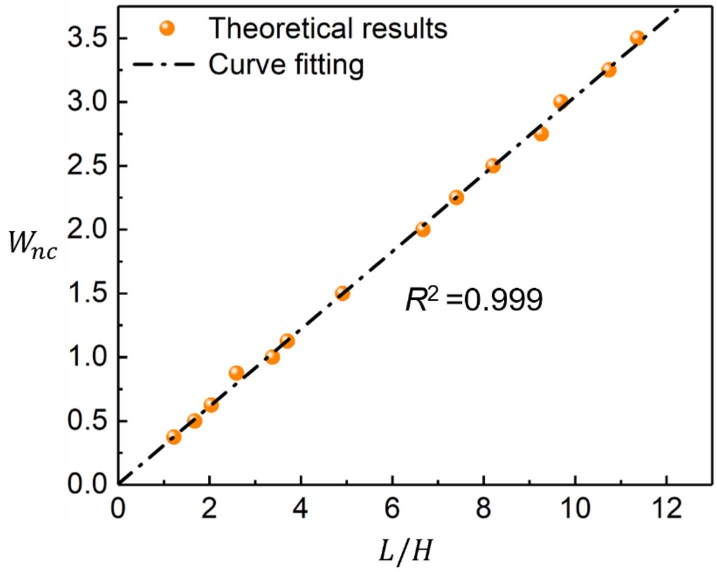


**Supplementary Fig. 24. Theoretical results and fitting for the relationship between $W_{\mathrm{nc}}$ and the aspect ratio (*L/H*).** The dashed line represents the linear fitting result with $R^2$ of 0.999.

**Supplementary Table 1. Comparison of the pumping efficiency with literature**

| Refs | Actuation strategy | *f* (Hz) | Medium | *Re* | *Q* (ul/min) | *P* (W) | *V* ($mm^3$) | $\varepsilon_{ef}$ |
|---|---|---|---|---|---|---|---|---|
| 8 | Magnetic actuation shape-memory pump | 270 | Glycerol solution | NA | 1800 | 0.208 | 25.0 | 92.9 |
| 9 | Magnetically actuated peristaltic motion | 20 | Water | NA | 74.7 | 0.016 | 78.4 | 0.07 |
| 10 | Chamber deformation via electromagnetic actuation | 1~20 | Water | NA | 67.5 | 0.049 | 24.7 | 0.06 |
| 4 | Cilium motion | 31.8 | Water | NA | $2.1\times10^{-14}$ | $2.5\times10^{-14}$ | $5.4\times10^{-10}$ | 30.4 |
| 11 | Cilia array inspired motion | 2.5 | Glycerol solution | 0.03 | 150 | NA | NA | NA |
| 6 | Magnetically actuated centrifugal pump | 1.6~16.6 | Water and fat emulsion | NA | 30600 | 4.1 | NA | NA |
| This work | Magnetically actuated dynamic transformation | 5 | Glycerol solution | 0.004 | 25.6 | 0.0014 | 25.9 | 262.6 |

Note: *f*: Actuation frequency; *Q*: Volume flow rate; *P*: Power; *V*: Volume of the actuator; $\varepsilon_{ef}$: Pumping efficiency (Dimensionless).

**Supplementary References**


1 Jiang, R., Xiao, J. & Song, J. Buckling of thin gel strip under swelling. Theoretical and Applied Mechanics Letters **7**, 134-137 (2017).

2 Kang, S. H. et al. Buckling-induced reversible symmetry breaking and amplification of chirality using supported cellular structures. Advanced materials **25**, 3380-3385 (2013).

3 Zhao, R., Kim, Y., Chester, S. A., Sharma, P. & Zhao, X. Mechanics of hard-magnetic soft materials. Journal of the Mechanics and Physics of Solids **124**, 244-263 (2019).

4 Eloy, C. & Lauga, E. Kinematics of the most efficient cilium. Physical Review Letters **109**, 038101 (2012).

5 Osterman, N. & Vilfan, A. Finding the ciliary beating pattern with optimal efficiency. Proceedings of the National Academy of Sciences **108**, 15727-15732 (2011).

6 Zhou, M. et al. Miniaturized soft centrifugal pumps with magnetic levitation for fluid handling. Science advances **7**, eabi7203 (2021).

7 Ren, Z., Hu, W., Dong, X. & Sitti, M. Multi-functional soft-bodied jellyfish-like swimming. Nature communications **10**, 1-12 (2019).

8 Smith, A. R., Saren, A., Järvinen, J. & Ullakko, K. Characterization of a high-resolution solid-state micropump that can be integrated into microfluidic systems. Microfluidics and Nanofluidics **18**, 1255-1263 (2015).

9 Saren, A., Smith, A. & Ullakko, K. Integratable magnetic shape memory micropump for high-pressure, precision microfluidic applications. Microfluidics and Nanofluidics **22**, 1-10 (2018).

10 Rusli, M., Chee, P. S., Arsat, R., Lau, K. X. & Leow, P. L. Electromagnetic actuation dual-chamber bidirectional flow micropump. Sensors and Actuators A: Physical **282**, 17-27 (2018).

11 Dong, X. et al. Bioinspired cilia arrays with programmable nonreciprocal motion and metachronal coordination. Science advances **6**, eabc9323 (2020).
