# Supplementary material for: Dynamic morphological transformations in soft architected materials via buckling instability encoded heterogeneous magnetization
Source: Nat Commun. 2022 Dec 6;13:7514. doi: 10.1038/s41467-022-35212-6 (PMC9727123; doi:10.1038/s41467-022-35212-6)
Supplement: Supplementary file 3 — Description of Additional Supplementary Files [file 41467_2022_35212_MOESM3_ESM.pdf]

Description of Additional Supplementary Files

File Name: Supplementary Movie 1
Description: Dynamic transformation of strip structure

File Name: Supplementary Movie 2
Description: Coupling stimulation on the strip structure

File Name: Supplementary Movie 3
Description: Morphological transformation of square lattice

File Name: Supplementary Movie 4
Description: Fluidic behaviors induced by strip structures

File Name: Supplementary Movie 5
Description: Reversible fluidic manipulation

File Name: Supplementary Movie 6
Description: Merging and transporting of droplet

File Name: Supplementary Movie 7
Description: Selective particle trap and release

File Name: Supplementary Movie 8
Description: Sensitivity-enhanced biomedical analysis

File Name: Supplementary Movie 9
Description: Efficient mixing of viscous fluid

File Name: Supplementary Movie 10
Description: Untethered swimming robot
