# Supplementary material for: Dynamic morphological transformations in soft architected materials via buckling instability encoded heterogeneous magnetization
Source: Nat Commun. 2022 Dec 6;13:7514. doi: 10.1038/s41467-022-35212-6 (PMC9727123; doi:10.1038/s41467-022-35212-6)
Supplement: Supplementary file 14 — Reporting Summary [file 41467_2022_35212_MOESM14_ESM.pdf]

nature portfolio

Corresponding author(s): Dongdong Jin, Li Zhang

Last updated by author(s): Nov 13, 2022

Reporting Summary

Nature Portfolio wishes to improve the reproducibility of the work that we publish. This form provides structure for consistency and transparency in reporting. For further information on Nature Portfolio policies, see our Editorial Policies and the Editorial Policy Checklist.

Statistics

For all statistical analyses, confirm that the following items are present in the figure legend, table legend, main text, or Methods section.

n/a	Confirmed	
☐	☒	The exact sample size (*n*) for each experimental group/condition, given as a discrete number and unit of measurement
☐	☒	A statement on whether measurements were taken from distinct samples or whether the same sample was measured repeatedly
☒	☐	The statistical test(s) used AND whether they are one- or two-sided<br>*Only common tests should be described solely by name; describe more complex techniques in the Methods section.*
☒	☐	A description of all covariates tested
☒	☐	A description of any assumptions or corrections, such as tests of normality and adjustment for multiple comparisons
☐	☒	A full description of the statistical parameters including central tendency (e.g. means) or other basic estimates (e.g. regression coefficient) AND variation (e.g. standard deviation) or associated estimates of uncertainty (e.g. confidence intervals)
☒	☐	For null hypothesis testing, the test statistic (e.g. *F*, *t*, *r*) with confidence intervals, effect sizes, degrees of freedom and *P* value noted<br>*Give P values as exact values whenever suitable.*
☒	☐	For Bayesian analysis, information on the choice of priors and Markov chain Monte Carlo settings
☒	☐	For hierarchical and complex designs, identification of the appropriate level for tests and full reporting of outcomes
☒	☐	Estimates of effect sizes (e.g. Cohen's *d*, Pearson's *r*), indicating how they were calculated

Our web collection on statistics for biologists contains articles on many of the points above.

Software and code

Policy information about availability of computer code

Data collection	The simulation of the magnetic field distribution and the coupling stimulation of magnetic field and solvent was performed by COMSOL Multiphysics 5.5.<br>The simulation of the transformation under magnetic stimuli was conducted by ABAQUS 2016.<br>The actuation of the magnetic soft robot was performed with LABVIEW 2017.
Data analysis	MATLAB R2019a

For manuscripts utilizing custom algorithms or software that are central to the research but not yet described in published literature, software must be made available to editors and reviewers. We strongly encourage code deposition in a community repository (e.g. GitHub). See the Nature Portfolio guidelines for submitting code & software for further information.

Data

Policy information about availability of data

All manuscripts must include a data availability statement. This statement should provide the following information, where applicable:

- Accession codes, unique identifiers, or web links for publicly available datasets
- A description of any restrictions on data availability
- For clinical datasets or third party data, please ensure that the statement adheres to our policy

All data generated in this study are provided in the article and its Supplementary Information.

Human research participants

Policy information about studies involving human research participants and Sex and Gender in Research.

Reporting on sex and gender	No human research participants were involved.
Population characteristics	Not relevant.
Recruitment	Not relevant.
Ethics oversight	Not relevant.

Note that full information on the approval of the study protocol must also be provided in the manuscript.

Field-specific reporting

Please select the one below that is the best fit for your research. If you are not sure, read the appropriate sections before making your selection.

☐ Life sciences ☐ Behavioural & social sciences ☐ Ecological, evolutionary & environmental sciences

For a reference copy of the document with all sections, see nature.com/documents/nr-reporting-summary-flat.pdf

Life sciences study design

All studies must disclose on these points even when the disclosure is negative.

Sample size	No Life sciences study design was involved.
Data exclusions	Not relevant.
Replication	Not relevant.
Randomization	Not relevant.
Blinding	Not relevant.

Behavioural & social sciences study design

All studies must disclose on these points even when the disclosure is negative.

Study description	Not relevant.
Research sample	Not relevant.
Sampling strategy	Not relevant.
Data collection	Not relevant.
Timing	Not relevant.
Data exclusions	Not relevant.
Non-participation	Not relevant.
Randomization	Not relevant.

Ecological, evolutionary & environmental sciences study design

All studies must disclose on these points even when the disclosure is negative.

Study description	Not relevant.
Research sample	Not relevant.
Sampling strategy	Not relevant.
Data collection	Not relevant.
Timing and spatial scale	Not relevant.
Data exclusions	Not relevant.
Reproducibility	Not relevant.
Randomization	Not relevant.
Blinding	Not relevant.

Did the study involve field work? ☐ Yes ☒ No

Reporting for specific materials, systems and methods

We require information from authors about some types of materials, experimental systems and methods used in many studies. Here, indicate whether each material, system or method listed is relevant to your study. If you are not sure if a list item applies to your research, read the appropriate section before selecting a response.

Materials & experimental systems

n/a	Involved in the study
☒	☐ Antibodies
☒	☐ Eukaryotic cell lines
☒	☐ Palaeontology and archaeology
☒	☐ Animals and other organisms
☒	☐ Clinical data
☒	☐ Dual use research of concern

Methods

n/a	Involved in the study
☒	☐ ChIP-seq
☒	☐ Flow cytometry
☒	☐ MRI-based neuroimaging
